# Genetic Characterization of Coronaviruses from Domestic Ferrets, Japan

**DOI:** 10.3201/eid2002.130543

**Published:** 2014-02

**Authors:** Yutaka Terada, Shohei Minami, Keita Noguchi, Hassan Y.A.H. Mahmoud, Hiroshi Shimoda, Masami Mochizuki, Yumi Une, Ken Maeda

**Affiliations:** Yamaguchi University, Yamaguchi, Japan (Y. Terada, S. Minami, K. Noguchi, H.Y.A.H. Mahmoud, H. Shimoda, K. Maeda);; Kagoshima University, Kagoshima, Japan (M. Mochizuki);; Azabu University, Kanagawa, Japan (Y. Une)

**Keywords:** coronaviruses, ferret, domestic ferret, genotype, Japan, genetic characterization, pets, viruses

## Abstract

We detected ferret coronaviruses in 44 (55.7%) of 79 pet ferrets tested in Japan and classified the viruses into 2 genotypes on the basis of genotype-specific PCR. Our results show that 2 ferret coronaviruses that cause feline infectious peritonitis–like disease and epizootic catarrhal enteritis are enzootic among ferrets in Japan.

An epizootic catarrhal enteritis (ECE) was first recognized in domestic ferrets (*Mustelo putorius furo*) in the United States in 2000 (*1*). The causative agent of ECE was demonstrated to be a novel ferret coronavirus (FRCoV) belonging to the genus *Alphacoronavirus* (*1*,*2*). Ferrets with ECE showed general clinical signs of lethargy, anorexia, and vomiting and had foul-smelling, green mucous–laden diarrhea. A systemic infection of ferrets closely resembling feline infectious peritonitis (FIP) was subsequently reported among ferrets in the United States and Europe. The causative agent was also shown to be an *Alphacoronavirus*, which was named ferret systemic coronavirus (FRSCV) (*3*,*4*); this virus was found to be genetically distinct from those associated with ECE and from 2 viruses assigned to different genotypes (*5*). Other cases of ECE and ferret infectious peritonitis have since been described in the United States and in Europe (*2*–*4*,*6*,*7*). One case of pathology-confirmed FIP-like disease has been described among domestic ferrets in Japan (*8*). The goal of this study was to determine the prevalence of coronavirus among domestic ferrets seen by veterinarians in various parts of Japan.

## The Study

Fecal samples were collected during August 2012–July 2013 from 79 ferrets from 10 animal hospitals scattered across 5 prefectures in Japan. Most of the ferrets were brought to veterinarians for clinical signs such as diarrhea, abdominal masses, and hypergammaglobulinemia; some had signs unrelated to coronavirus infection or were asymptomatic (Table 1). The diarrhea tended to be mild, unlike with ECE, and was found in coronavirus-negative and -positive animals. 

**Table 1:** Detection of FRCoV from ferrets with clinical signs, Japan

Sample type	No. (%) samples			
	Diarrhea, n = 34	Hypergammaglobulinemia, n = 6	Abdominal mass, n = 14	Nonrelated signs/ asymptomatic, n = 33
All FRCoV-positive samples†	25 (73.5)	5 (83.3)	7 (50.0)	17 (51.5)
Genoype I samples‡	17 (50.0)	2 (33.3)	4 (28.6)	10 (30.3)
Genoype II samples§	7 (20.6)	1 (16.7)	4 (28.6)	7 (21.2)

*FRCoV, ferret coronavirus; RT-PCR, reverse transcription PCR. †RT-PCR was carried out by using FRCoV-specific primers. ‡RT-PCR was carried out by using type 1 FRCoV-specific primers (*5*). §RT-PCR was carried out by using type 2 FRCoV-specific primers (*5*).

**Figure 1:**
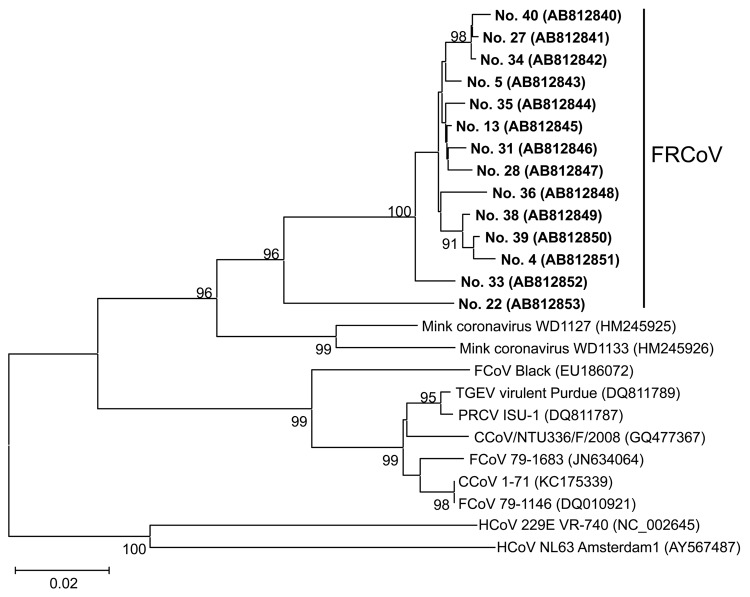
Phylogenetic tree constructed on the basis of the nucleotide sequences of the partial RNA-dependent RNA polymerase–encoding regions of ferret coronaviruses (FRCoVs) isolated in Japan (shown in boldface; sample IDs are indicated) compared with other coronaviruses (CoVs). The tree was constructed by the neighbor-joining method in MEGA5.0 software (*10*); bootstrap values of >90 are shown. DDBJ/EMBL-Bank/GenBank accession numbers for the nucleotide sequences are shown in parentheses. Human CoVs (HCoVs) 229E and NL63, which belong to the *Alphacoronavirus* genus, were used as the outgroup. CCoV, canine coronavirus; FCoV, feline coronavirus; TGEV, transmissible gastroenteritis virus; PRCoV, porcine respiratory coronavirus. Scale bar indicates nucleotide substitutions per site.

RNA was extracted from fecal samples by using the QIAamp Viral RNA Mini Kit (QIAGEN, Hilden, Germany), and reverse transcription PCR (RT-PCR) was performed by using the QIAGEN OneStep RT-PCR Kit (QIAGEN) using coronavirus consensus primers IN-6 and IN-7, which amplify the open reading frame (ORF) 1b region, encoding RNA-dependent RNA polymerase (RdRp). This primer pair can amplify nucleic acids from many coronaviruses in the subfamily *Coronavirinae* (*9*). Of 79 samples, 33 (41.8%) were positive for coronaviruses by RT-PCR (Table 2). Nucleotide sequences were determined for the amplified fragments and used to construct a phylogenetic tree (Figure 1). The coronaviruses detected in this study belonged to the genus *Alphacoronavirus* but formed a separate species from those of other species. The identities with feline coronavirus, transmissible gastroenteritis virus, porcine respiratory coronavirus, and mink coronavirus were 73.5%–75.9%, 73.5%–76.1%, 73.8%–76.1%, and 80.2%–84.0%, respectively.

**Table 2:** Comparison of results for detection of FRCoV in ferret fecal samples by RT-PCR using coronavirus consensus and FRCoV-specific primers, Japan

Coronavirus consensus primers	FRCoV-specific primers		Total no. (%)
	No. positive samples	No. negative samples	
No. positive samples	31	2	33 (41.8)
No. negative samples	13	33	46 (58.2)
Total no. (%)	44 (55.7)	35 (44.3)	79

*FRCov, ferret coronavirus; RT-PCR, reverse transcription PCR.

On the basis of additional sequence data, a new primer pair was designed: forward FRCoV RdRp-F1 (5′-GTT GGT TGC TGC ACA CAT AG-3′) and reverse FRCoV RdRp-R1 (5′-GGA GAA GTG CTT ACG CAA ATA-3′). Results for RT-PCR using this new primer set showed that 44 (55.7%) of 79 samples were positive for coronavirus, which was a higher number than that obtained by using the published coronavirus consensus primers (55.7% vs. 41.8%) (Table 2). Two samples that had positive results by consensus primers had negative results by the new primers: sample 22 had many mutations in the primer binding site (Figure 1), whereas sample 40 had few mutations.

**Figure 2:**
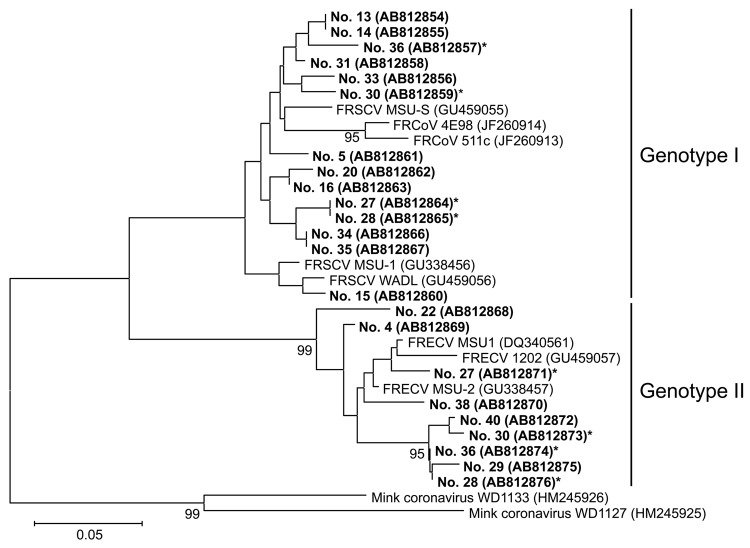
Phylogenetic tree based on the nucleotide sequences of partial S genes of ferret coronaviruses (FRCoVs) isolated in Japan (shown in boldface; sample IDs are indicated) compared with other coronaviruses (CoVs). The tree was constructed by the neighbor-joining method in MEGA5.0 software (*10*); bootstrap values of >90 are shown. Asterisks indicate samples from ferrets infected with FRCoVs of both genotypes1 and 2. DDBJ/EMBL-Bank/GenBank accession numbers for the nucleotide sequences are shown in parentheses. FRSCV, ferret systemic coronavirus; FRECV, ferret enteric coronavirus. Scale bar indicates nucleotide substitutions per site.

On the basis of the partial sequences of the spike gene, Wise et al. (*5*) reported that the known ferret coronaviruses could be divided into 2 genotypes: genotype 1, which included the agent of FIP-like disease, and genotype 2, which included the causative agent of ECE. To differentiate between these genotypes in the positive samples from our testing, RT-PCR was carried out by using 2 pairs of genotype-specific primers: forward primer 5′-CTG GTG TTT GTG CAA CAT CTA C-3′ and reverse primer 5′-TCT ATT TGC ACA AAA TCA GAC A-3′ for genotype 1, and forward primer 5′-GGC ATT TGT TTT GAT AAC GTT G-3′ and reverse primer 5′-CTA TTA ATT CGC ACG AAA TCT GC-3′ for genotype 2 (*5*). Among these ferrets, 30 (38.0%) were infected with genotype 1 and 17 (21.5%) with genotype 2; 8 (10.1%) ferrets were infected with both genotypes of coronaviruses (Figure 2). Samples 27 and 28 were from ferrets that lived in the same house and harbored the same ferret coronavirus but that were born on different farms, indicating that horizontal transmission had occurred. The nucleotide sequences of the amplified genes confirmed that these coronaviruses also fell into genotypes 1 and 2 (Figure 2). 

Our results indicate that both genotypes of coronavirus have been spreading within the ferret population in Japan for some time, and some ferrets have been coincidentally infected with both genotypes. Of note, most ferrets that were positive for genotype 1 ferret coronavirus in this study did not show FIP-like disease (Table 1), indicating that infection with genotype 1 ferret coronavirus does not always cause FIP-like disease. Genotype 1 ferret coronavirus has also been detected from asymptomatic ferrets in the Netherlands (*11*).

To further investigate virus transmission routes, oral swab specimens were collected from 14 of the 79 ferrets and examined by RT-PCR using primers FRCoV RdRp-F1 and FRCoV RdRp-R1. Five (35.7%) specimens were positive (data not shown), providing a route leading to infection of susceptible animals. Coronaviruses are known to cause both respiratory and intestinal diseases in various animal species; therefore, ferret coronaviruses should be investigated in respiratory disease.

## Conclusions

We established a sensitive RT-PCR method using a new primer pair to detect coronavirus sequences and demonstrated that ferret coronaviruses are widespread among ferrets in Japan. We determined the partial nucleotide sequences of the spike gene of 23 strains and found they were clearly divided into 2 genotypes, 1 and 2 (Figure 2). The reported ferret coronaviruses associated with FIP-like disease, designated as genotype 1 by Wise et al. (*5*), all fell within genotype 1 phylogenetically, whereas all published ECE-causing strains fell within genotype 2. This finding leads to a possible conclusion that FIP-like disease–causing strains (i.e., FRSCVs) are variants of what has been designated genotype 1 ferret coronaviruses. Because we found no relationship between the 2 genotypes of ferret coronavirus and the type of disease (Table 1), we cannot determine whether FIP-like and ECE-like ferret coronaviruses circulate independently as distinct entities or evolve, like feline coronaviruses, from more ubiquitous and less pathogenic enzootic strains. Nonetheless, the addition of these 23 new isolates to the phylogenetic tree of ferret coronaviruses tends to support the latter conclusion. Without extensive animal passage studies, virus isolation, and coronavirus-free ferrets, this theory may be difficult to confirm. However, additional evidence tends to link virulent pathotypes of ferret coronaviruses to specific mutational events. Nucleotide sequences of the 3c-like protein genes of FRSCV, MSU-1 (DDBJ/EMBL-Bank/GenBank accession no. GU338456), MSU-S (GU459059), and WADL (GU459058), showed that 2, MSU-1 and WADL, possessed a truncated 3c-like protein gene (*5*), similar to that described for FIP viruses of cats (*12*–*14*). FIP-causing viruses of cats also contain a second mutation in the spike gene (*15*), which was not investigated in our study. The existence of 2 major genotypes of Japanese ferret coronaviruses is also reminiscent of the serotype I and II feline coronaviruses. Without ferret coronaviruses that can be grown in cell culture, however, such serologic differentiation will be difficult.
